# Conjugated Polymer Nanoparticles for Bioimaging

**DOI:** 10.3390/ma10121420

**Published:** 2017-12-12

**Authors:** Yasmine Braeken, Srujan Cheruku, Anitha Ethirajan, Wouter Maes

**Affiliations:** 1 Institute for Materials Research (IMO-IMOMEC), Design & Synthesis of Organic Semiconductors (DSOS), UHasselt—Hasselt University, Agoralaan, 3590 Diepenbeek, Belgium; yasmine.braeken@uhasselt.be; 2 Associated Lab IMOMEC, IMEC, Wetenschapspark 1, 3590 Diepenbeek, Belgium; srujan.cheruku@uhasselt.be; 3 Institute for Materials Research (IMO-IMOMEC), Nanobiophysics and Soft Matter Interfaces (NSI), UHasselt—Hasselt University, Agoralaan, 3590 Diepenbeek, Belgium

**Keywords:** conjugated polymers, nanoparticles, fluorescence, bioimaging

## Abstract

During the last decade, conjugated polymers have emerged as an interesting class of fluorescence imaging probes since they generally show high fluorescence brightness, high photostability, fast emission rates, non-blinking behavior and low cytotoxicity. The main concern related to most conjugated polymers is their lack of hydrophilicity and thereby poor bio-availability. This can, however, be overcome by the formulation of conjugated polymer nanoparticles in aqueous medium. This review provides an overview of the different techniques employed for the preparation of conjugated polymer nanoparticles, together with methods to improve their photoluminescence quantum yields. For selective targeting of specific cells, dedicated surface functionalization protocols have been developed, using different functional groups for ligand immobilization. Finally, conjugated polymer nanoparticles have recently also been employed for theranostic applications, wherein the particles are simultaneously used as fluorescent probes and carriers for anti-tumor drugs.

## 1. Introduction

Bioimaging is a powerful method to gain insights in biological processes and malfunctions [1]. Over the past decades, several techniques were developed to create images of organs, veins and cells, such as magnetic resonance imaging (MRI) [2], computerized tomography (CT) [3], ultrasound imaging [4] and positron emission tomography (PET) [5]. Nevertheless, the search for improved cost-effective, time dependent and safe bioimaging techniques with an excellent resolution is still ongoing. Fluorescence imaging allows the visualization of biological processes from the cellular down to the molecular level in an easy and non-destructive way [6]. As a result, fluorescence-based diagnosis of diseases and fluorescence image guided surgery have been shown to be successful applications [7].

Different types of emissive bioimaging probes have been reported. Fluorescent organic dyes can exhibit high photoluminescence quantum yields (PLQYs) and a large variety of dyes with tunable optical characteristics are readily available. However, those small organic molecules generally exhibit a low (photo)stability and photobleaching often presents a problem [8,9,10,11]. The stability of the fluorescent probes can be improved by the use of inorganic quantum dots, consisting of heavy metals like lead, cadmium or indium, but the presence of those metals significantly increases the cytotoxicity [8,12,13]. Conjugated polymers (CPs) have recently gained considerable interest as they are generally stable and non-cytotoxic and their structure can be readily adapted to tune the optical characteristics [14,15]. CPs have a backbone of alternating σ- and π-bonds, inducing semi-conductivity. The bandgap of the polymer strongly depends on its composition. In the field of organic electronics, CPs have been studied extensively over the past decades. The introduction of an alternating ‘push–pull’ or ‘donor-acceptor’ motif has been used frequently to lower the bandgap [16]. Typical push/donor entities are electron-rich monomers with high-lying energy levels, whereas pull/acceptor moieties have low-lying energy levels. This concept allows for stretching the absorption and emission spectra of CPs as far as the near infra-red (NIR) region. This wavelength range is attractive for bioimaging, as it enables deep tissue penetration and minimal background autofluorescence. Moreover, low energy optical waves are non-destructive for tissues [17].

However, for applications in biological environments, it is imperative that the fluorescent probes are water-soluble. Since CP polymer backbones are generally hydrophobic, strategies have to be implemented to make them operable in aqueous media. One particular approach uses the introduction of charged moieties on the polymer side chains, creating conjugated polyelectrolytes (CPEs) [18,19]. These CPEs are soluble in water and can hence be used individually as fluorescent probes. Other strategies link the CP to biological structures such as human serum albumin (HSA) or liposomes [20,21]. A more common technique is to prepare a polymer dispersion in water or a buffer solution. Small conjugated polymer nanoparticles (CPNPs), also known as polymer dots (Pdots), are thus created, stabilized by surfactant molecules [22,23,24].

When developing CPNPs, one must keep in mind that the optical properties of the polymer change depending on the size of the particles [25] and the conjugation length [26]. CPs formulated into NPs exhibit similar optical properties as bulk thin-films and these features can differ significantly from those observed in a good solvent. Another complication induced by the tight packing of the polymer chains in the particles is fluorescence quenching, which significantly decreases the PLQY and the brightness of the probe. Excited state reactions, resonance energy transfer (Förster and Dexter mechanisms), intersystem crossing, collisional quenching (mainly in the gas state and in solution), photoinduced electron transfer (PET) and ground state complex formation are typical fluorescence quenching processes that have been described in literature. The quenching process can differ from one system to another and often different mechanisms are playing at the same time [27,28,29,30].

In this review, the focus lies on recent (chemical) developments in bioimaging based on CPNPs. The applied protocols for the improvement of the PLQY through reduction of quenching processes are discussed. Furthermore, some strategies employed for surface functionalization and cell targeting are highlighted.

## 2. Recent Developments in Bioimaging with CPNPs

### 2.1. Preparation of Conjugated Polymer Nanoparticles

**Figure 1:**
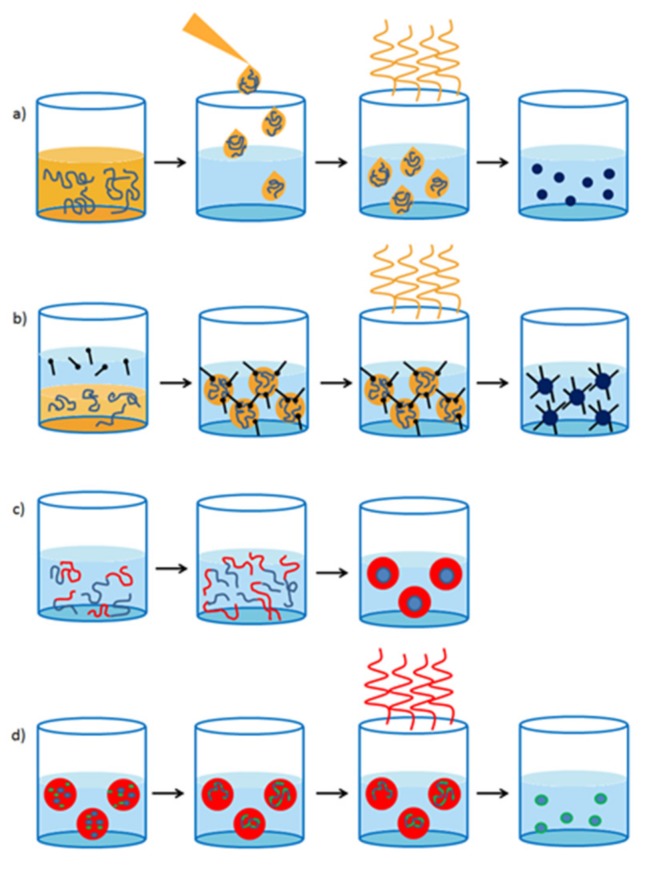
Conjugated polymer nanoparticle formulation via (**a**) the solvent exchange technique; (**b**) the mini-emulsion technique; (**c**) self-assembly and (**d**) emulsion polymerization.

Different techniques have been employed for the synthesis of CPNPs. The solvent exchange method is most frequently used (Figure 1a). In this procedure, the CP is dissolved in a good, water miscible solvent, e.g., tetrahydrofuran (THF). The polymer solution is subsequently added into water while sonicating. When the polymer solution is added to the water phase, the solubility of the polymer drops drastically and the polymer precipitates in very small particles. As such, this technique is also often referred to as the nanoprecipitation method [31,32]. Afterwards, the organic solvent residues are removed by evaporation and the CPNPs remain dispersed in water. In general, particles smaller than 40 nm are formed by this approach and the size can be tuned by the polymer concentration, water temperature and solubility of the polymer. CPNPs are less commonly prepared via the mini-emulsion technique (Figure 1b). Here, a continuous phase and a dispersed phase are combined. The former consists out of a surfactant dissolved in water and the latter contains the polymer in a water-immiscible solvent, e.g., chloroform. By applying strong shear forces via ultra-sonication to the two-phase system, the dispersed phase is bursting into small droplets containing the CP and packed by the surfactant on the outside. The size of the nanoparticles can be adjusted by varying the polymer:surfactant ratio and typically ranges from 40 to 500 nm. In addition in this method, the water-immiscible organic solvent is removed via evaporation in the final step. Even less employed techniques are the self-assembly method (Figure 1c), in which the polymers assemble into predefined structures due to specific molecular interactions [33], and the emulsion polymerization technique (Figure 1d), in which the polymer is readily synthesized in preformed emulsion droplets [22,34]. In 2014, Yoon et al. [35] developed a new nanoparticle formation technique in which phase-separated films of CPs and phospholipids were split up in particles by sonication. However, this technique is not considered as a standard particle synthesis method. Table 1 provides an overview of the preparation methods of the particles discussed further on in this review article.

### 2.2. Strategies to Implement CPNPs for Bioimaging

**Figure 2:**
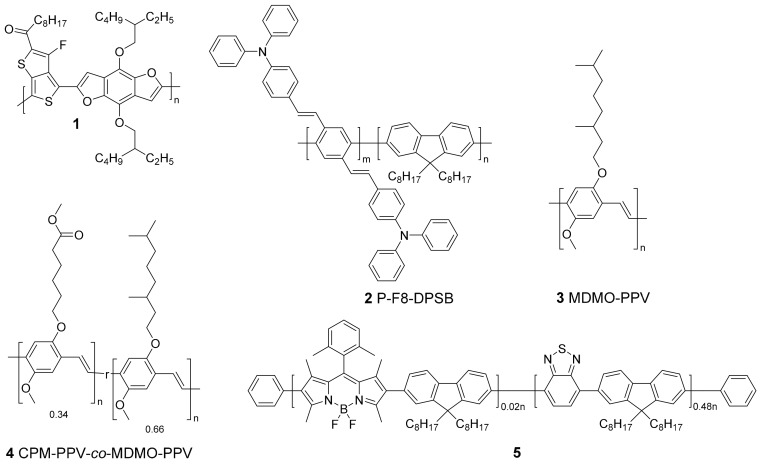

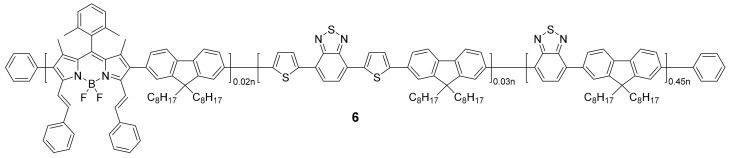
Structures of conjugated polymers with absorption in the near-infrared region because of the strong donor-strong acceptor approach (**1**), two-photon excitation (**2**–**4**) and extra narrow emission peaks (**5**, **6**).

Fluorescent dyes are often added in a few mass percent to non-conjugated polymer or silica matrices to form fluorescent nanoparticles. Nevertheless, problems concerning leaching of the dye out of the matrix and poor photostability have stimulated research into alternative strategies. By replacing typical small molecule fluorescent dyes by conjugated polymers, leaching can be overcome. Furthermore, since the polymers themselves serve as fluorescent probes, no external matrix material is needed anymore to fixate the probe in the particles. During the last decade, many different CPs have been studied as fluorescent imaging probes. Highly complicated polymer structures with uncommon monomer moieties, nowadays developed for organic electronics, are not widely used in the bioimaging field. The polymer backbone structures are overall simple and easy to synthesize. Poly(*p*-phenylene ethynylene)s (PPEs) and poly(*p*-phenylene vinylene)s (PPVs) are typical examples of such polymers. However, since their emission spectrum does not reach into the attractive first NIR window (650–1000 nm), push–pull conjugated polymers are also emerging in the bioimaging field. The most often employed polymers are based on fluorene, copolymerized together with benzothiadiazole, quinoxaline and/or thiophene. The influence of monomer ratios and side chain variations on the optical properties, particle formation, stability and in vitro/in vivo imaging have been widely studied (*vide infra*). Hong et al. [36] pushed the emission of their NPs into the second NIR window (1000–1350 nm) by developing a push–pull conjugated polymer (**1**, Figure 2) based on a strong fluorinated thieno[3,4-*b*]thiophene acceptor. The polymer was formed into small particles via the mini-emulsion technique and the CPNPs were stabilized with a PEGylated (PEG = polyethylene glycol) surfactant. While the absorption maximum was found at λ = 654 nm, an impressive Stokes shift of 400 nm was observed, with an emission peaking at λ = 1047 nm. This long emission wavelength is beneficial for bioimaging because of a lower autofluorescence and reduced photon scattering in biological tissues, resulting in a higher spatial resolution and deeper tissue penetration. A drawback of CPNPs with emission peaks in the second optical window is the decrease in PLQY. CPNPs synthesized from CP **1** exhibit a poor PLQY of 1.7%, which might be high for fluorophores emitting in this long wavelength region, but low in comparison to emitters in the first NIR window (Table 1). Hong and coworkers were, however, able to monitor arterial blood flow in vivo due to the excellent time resolution (20 ms) that could be obtained with these particles [36]. Since the cardiac cycle in mice takes 200 ms, changes in blood velocity during this cycle could be observed. Furthermore, the outstanding spatial resolution obtained with those CPNPs enabled tracking of blood flow in capillary vessels with a sub-10 µm diameter, which had not been realized before with traditional ultrasound and optical coherence tomography (OCT). This real-time haemodynamic imaging can be of high importance to improve our understandings of cardiovascular diseases and to design treatments accordingly.

Recently, more interest has also gone into two-photon excitation microscopy, where a single excitation is the result of the simultaneous absorption of two-photons with longer wavelength. Because typical excitation wavelengths are in the NIR regime, the beam can penetrate deeper into tissue (1 mm) and it causes less damage to the biological tissue. The probability of emission increases drastically (nonlinear) when the excitation beam intensity is high. This means that scattered light does not contribute to the output signal, leading to a high optical resolution [37]. Most conjugated polymers have shown to be good two-photon excitation probes [38,39]. This was also illustrated by Lv et al. [40], who combined fluorene based CP **2** (Figure 2) with a perylene diimide (PDI) dye, creating particles that can be excited at λ = 800 nm, while emission occurs at λ = 730 nm. Peters et al. [41] also investigated two-photon excitation of their PPV-based NPs (**3** and **4**; Figure 2) and they were able to excite the particles at 830 nm, while the fluorescence maximum lies at 580 nm.

For some applications, multiple targets have to be detected simultaneously, which is referred to as spectral multiplexing. To realize this, probes with narrow emission peaks are required to prevent emission overlay. Rong and coworkers [42] developed boron dipyrromethene (BODIPY) based push–pull conjugated polymers (**5** and **6**; Figure 2) with emission peak widths at half maximum of only 40–55 nm, which is 1.5 to 2 times narrower than the emission peak widths of conventional CPNPs [9]. This can be achieved by efficient intra-particle energy transfer to the BODIPY units, which are known to be narrow-emissive species. This property is transferred onto the BODIPY containing polymers.

### 2.3. Improving the Photoluminescence Quantum Yield

**Figure 3:**
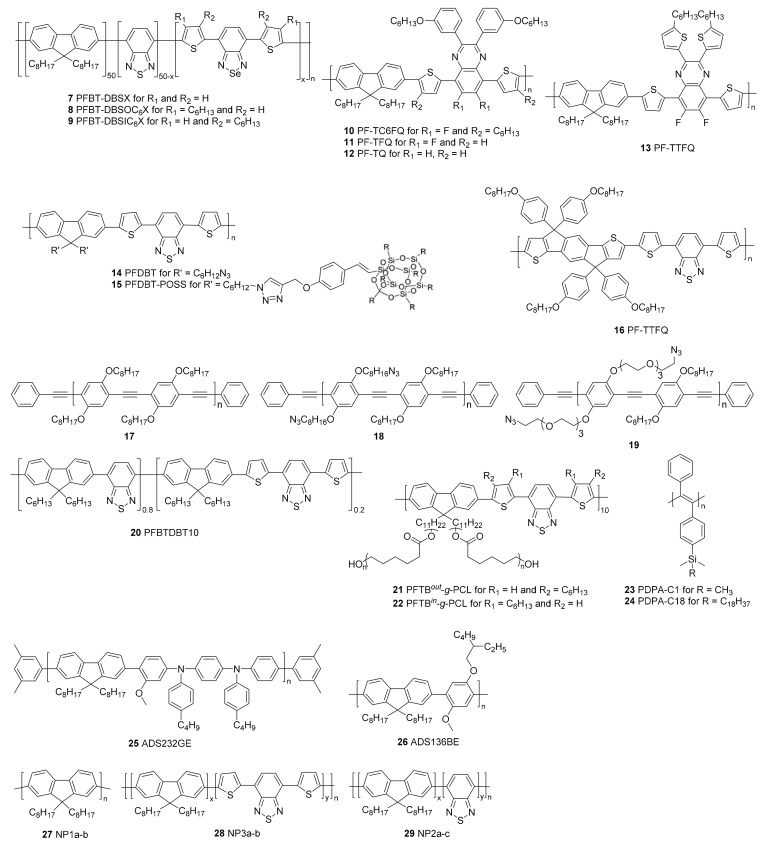
Conjugated polymers affording improved photoluminescence quantum yields in nanoparticle form.

All conjugated polymers discussed in this section are developed to increase the PLQYs and their structures are gathered in Figure 3. PLQYs of CPNPs are in general smaller than those of their molecularly dissolved CP counterparts. This can mainly be attributed to quenching processes due to the close proximity of multiple polymer chains.

**Figure 4:**
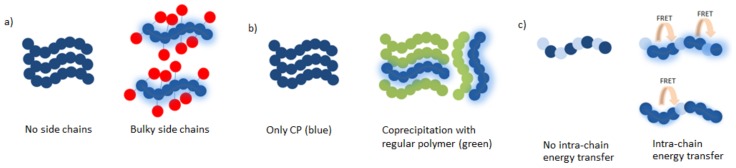
Different approaches to improve the photoluminescence quantum yield of conjugated polymer nanoparticles: (**a**) The use of bulky side chains; (**b**) the introduction of regular, non-conjugated polymers; and (**c**) varying monomer ratios.

The main strategy employed to diminish quenching is to increase the polymer inter-chain distances within one particle. Different approaches have been investigated but the most convenient method is to include (bulky) side chains onto the polymer backbone, which limit stacking by imposing steric hindrance (Figure 4a). Chen et al. [43] illustrated this approach by the introduction of hexyl side chains onto dithienylbenzoselenadiazole (DBS) based polymers **7**–**9**. Alkyl substitution on the thiophene subunits improved the quantum yield from 2% for the non-substituted derivative to 8% for polymer **9**, where the hexyl chains point toward the DBS unit. A maximum of 15% was even obtained for polymer **8**, where the side chains point outwards from DBS. Liu et al. [44] developed a series of quinoxaline based polymers **10**–**13**. Substitution of the phenyl rings on the quinoxaline 2,3-positions by thiophene moieties (**13**) reduced the PLQY from 11 to 8%. On the other hand, a red shift of both the absorption and emission spectra could be observed due to the extension of the conjugated system in the thiophene rings. In addition, the effect of substitution on the polymer backbone was investigated. The introduction of fluorine atoms on the quinoxaline 6,7-positions slightly raised the PLQY from 9 (**12**) to 11% (**11**). Also in this work, the introduction of hexyl side chains pointing outwards from the quinoxaline unit afforded a strong increase in PLQY, from 11% for polymer **11** to 47% for polymer **10**. The introduction of the bulky polyhedral oligomeric silsesquioxane (POSS) side chain increased the PLQY of poly{[9,9-di(hexyl)fluorene]-*alt*-[4,7-bis(thiophen-2′-yl)-2,1,3-benzothiazole]} (PFDBT, **14**) NPs from 2 to 14% (PFDBT-POSS **15**) [45]. A 4-octyloxyphenyl side chain was introduced by the same group onto the indacenothiophene units of polymer **16** [46]. The bulkiness of those side chains hinders efficient π–π stacking, which is beneficial for the fluorescence efficiency due to the inhibition of charge transfer induced fluorescence quenching.

In addition, smaller functional groups can have an influence on the quantum efficiency of CPNPs. This was illustrated by D’Olieslaeger et al. [47], who designed PPEs with azides on octyloxy (**18**) or tetra(ethylene glycol) (TEG) (**19**) side chains. The PLQYs of the resulting NPs increased from 8% for **17**, the reference PPE containing no azide groups, to 13% for **18** and **19**. The influence of the TEG side chains on the PLQY is minimal but almost no cell penetration was observed for the CPNPs consisting of **19**, which can be ascribed to low protein adsorption on the polymer surface. Furthermore, cell viabilities for the azide containing CPNPs were comparable to those of the particles devoid of azide moieties, indicating a non-toxic effect of the azide functionalization.

Co-precipitation of the CP with another polymer such as PEG can also isolate the polymer chains from each other, again preventing stacking and thus fluorescence quenching (Figure 4b). Co-precipitation of polymer **2** with a non-conjugated folic acid functionalized amphiphilic triblock copolymer was described by Lv et al. [40]. A bis(diphenylaminostyryl)benzene (DPSB) based CP (**2**) was developed to act as a FRET (Förster resonance energy transfer) donor in the CPNPs. A PDI dye was chosen as the acceptor material since it is emitting NIR light and the energy transfer efficiency from the donor to the acceptor material exceeds 90%. The co-precipitation of the different materials diminishes the stacking probability, together with the large DPSB groups and the octyl side chains on the fluorene subunit. The success of this approach is illustrated by a PLQY of 45% for the hybrid NPs. Ding et al. [48] also employed the co-precipitation technique to achieve a PLQY of 27% for CPNPs containing 50 mol % of **20** and 50 mol % of a non-conjugated PEG matrix material. Likewise, the formation of brush-like polymer structures can prevent aggregation of the CP chains. In this case, the brushes form a protecting outer layer around the CP backbone, isolating every single chain. Yang et al. [49] showed in their recent work that the introduction of polycaprolactone (PCL) side chains, acting as brushes, can improve the PLQY up to 5 times (to ~26% for **21**). The length of the PCL side chains had a small influence on the quantum efficiency, with the best PLQYs obtained for the longest PCL brushes. In addition, in this work, the effect of hexyl side chains on the thiophene rings was investigated and again the best results were obtained for polymer **22**, in which the hexyl groups point outwards.

A very promising technique was introduced by Kim et al. [50], who showed that the freezing of polydiphenylacetylene (PDPA, **23** and **24**) into CPNPs in their relaxed state can lead to an extremely high PLQY of 76%. PDPA is an amorphous polymer and the effect of relaxed state freezing is very specific for this polymer of which the backbone is rigid, but highly twisted because of the steric hindrance caused by the phenyl side chains. The twisted and sterically hindered structure can only undergo weak intermolecular interactions, meaning that intermolecular stacking is difficult. However, the phenyl rings can undergo intermolecular stacking, leading to fluorescence quenching in solid state films (PLQY of 1%). When **23** or **24** is dissolved in a good solvent like THF, the polymer chains become more flexible, reducing the intermolecular stacking and increasing the PLQY to 31%. However, in solution, collisional quenching and vibrational relaxation is still possible. Those quenching processes were reduced by freezing **23** in its relaxed state in CPNPs, leading to a PLQY of 76%. The quantum efficiency of CPNPs of **23** and **24** was compared to those of commercially available ‘highly emissive’ fluorene based polymers **25** and **26**, whose PLQY did not exceed 7%.

Behrendt et al. [51] tuned the quantum efficiency of fluorene based CPNPs by varying the amount of benzothiadiazole (BT) acceptor (Figure 4c). Polymer **28** was prepared with 5% and 10% of thiophene-BT-thiophene and the PLQYs were compared to the emission of **27**. In general, a decrease in the PLQY could be observed after the introduction of BT. However, the decrease was more pronounced for larger amounts of the acceptor unit. In polymer **29**, the introduction of 5–50% of BT was examined. A maximal PLQY of 56% was obtained for the 10% BT polymer, while lower amounts led to significantly lower PLQYs. This can be ascribed to a more effective FRET from fluorine–fluorene moieties to BT–fluorene units for higher BT amounts. The perfectly alternating donor–acceptor polymer showed the lowest PLQY, which was only 12%.

### 2.4. Surface Functionalization

**Figure 5:**
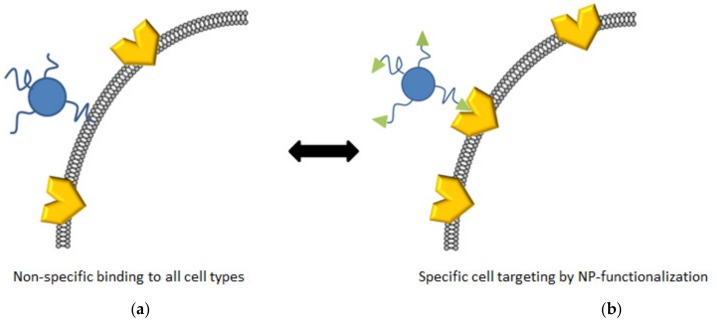
Non-specific CPNP–cell interactions (**a**) vs. specific cell targeting via the introduction of functionalities on the CPNPs that bind selectively to receptors present on the cells of interest (**b**).

**Figure 6:**
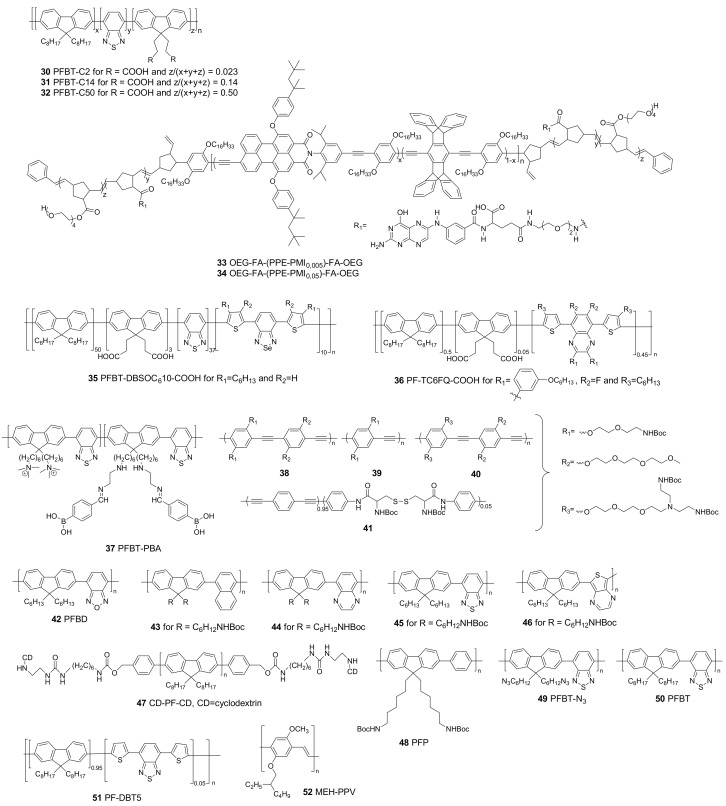
Functionalized CPNPs for covalent or non-covalent probe immobilization to target specific cells or organelles.

The functionalization of CPNPs can be a great asset to guide the particles to specific cells or organelles. For this purpose, probes that specifically bind receptors on the targeted cells can be covalently attached to the particle surface (Figure 5). Multiple strategies have been investigated to achieve such probe immobilization. All polymer structures discussed in this section are gathered in Figure 6. The most convenient technique is to functionalize the CP side chains, enabling straightforward covalent linking of the probe. The most widely used functional groups are carboxylic acid and *N*-hydroxysuccinimide (NHS) groups, allowing easy covalent linkage through the formation of amide bonds. In the case of the carboxylic acids, reaction with 1-ethyl-3-(3-dimethylaminopropyl)carbodiimide (EDC) results in O-acylisourea active esters. NHS, on the other hand, is already an activated ester. Both groups can then react with a primary amine to form the desired amide bond.

Zhang et al. [52] investigated the influence of the carboxylic acid side chain density of poly[(9,9-dioctylfluorenyl-2,7-diyl)-*co*-(1,4-benzo[2,1′,3]thiadazole)]s (PFBTs **30**–**32**) on particle formation, colloidal stability, internal structure, fluorescence brightness and non-specific cell adsorption. PFBT polymers with carboxylic acid molar fractions of 2.3%, 14% and 50% were synthesized. The PLQY of the CPNPs decreased with higher functionalization degrees, from 30% for polymer **30** to 17% for **32**. The same trend could be observed for the single particle fluorescence brightness (defined as the product of the extinction coefficient at the relevant wavelength and the PLQY). The non-specific adsorption was highest for the densely functionalized particles, while it was absent for particles prepared from **30**. The overall performance was best for the CPNPs synthesized from PFBT **30**. Those particles were then covalently bound to streptavidin and successful imaging of HER2-overexpressed breast cancer cells (SKBR-3) was achieved.

Ahmed and co-workers [53] also chose to introduce functional groups onto the polymer side chains. They developed a pentablock copolymer (**33** and **34**) of an ABCBA structure in which a fluorescent PPE core (block C) comprising 0.5 to 5% of perylene monoimide (PMI) is coupled to an NHS functionalized block (B). Block A is an oligo(ethylene glycol) structure to improve water solubility, stealth-like and anti-fouling properties. Since folate receptors (FR) are overexpressed on cell membranes of many different cancer cell types (e.g., in ovarian, breast, brain and lung cancer), the authors chose to covalently bind folate to the polymer side chains via NHS chemistry. After particle formation, the hydrophobic A block is situated in the core of the particles, whereas the hydrophilic blocks form a shell around the hydrophobic core, exposing the folate groups to the surrounding medium. The FA-functionalized CPNPs can be seen as a “Trojan Horse” because the activation of the FR induces endocytosis, leading to cancer cell internalization of the CPNPs. The particles with 5% PMI loading exhibited a lower PLQY of 14%, whereas the particles with only 0.5% of PMI showed a better quantum efficiency of 26%. Cell uptake of particles of **33** and **34** in KB cells (a sub-line of the HeLa tumor cell line) was three to six times higher compared to those of non-functionalized particles. Furthermore, at high concentrations, the cytotoxicity of the non-functionalized particles was higher due to the high reactivity of the NHS groups. Folate-functionalized NPs, on the other hand, showed no significant cell cytotoxicity.

The introduction of carboxylic acid functions onto the side chains of CPs (**35**) was also studied by Chen et al. [43], who were able to covalently bind streptavidin onto the CPNPs via EDC coupling. Streptavidin has an extremely high affinity for biotin, which enables the labelling of cellular and subcellular structures when biotinylated receptor ligands of interest are administered. Their hypothesis was confirmed by the imaging of subcellular microtubules in HeLa cells after incubation with a biotinylated monoclonal anti-α-tubulin antibody and by the imaging of MCF-7 cell membranes after incubation with biotinylated primary antihuman CD326 EpCAM antibody. The Pdots are able to bind the biotinylated receptor ligands selectively due to the presence of streptavidin. Moreover, no non-specific adsorption was observed in any of the studied cases.

**Figure 7:**
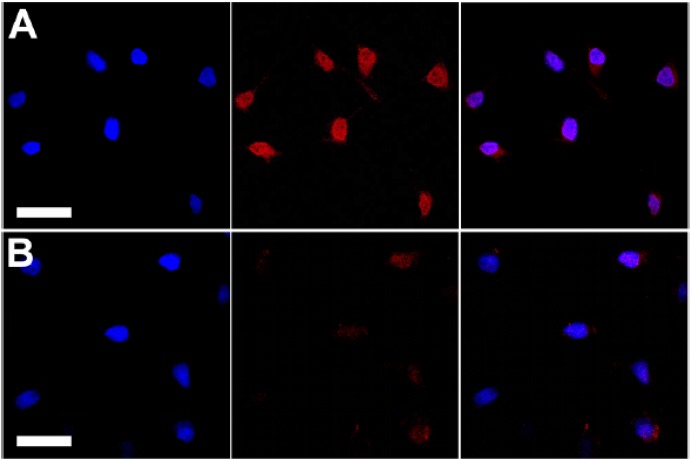
Confocal fluorescence images of SKOV-3 cells labeled by Pdot-folate conjugates (based on CP **36**) and the flow cytometry results using MCF-7 cells. (**A**) The blue fluorescence results from the nuclear counterstain Hoechst 34580, and the red fluorescence is due to the Pdot–folate conjugates. The right panel shows the overlay of the blue and red fluorescence; (**B**) images of negative control samples in which cells were incubated with bare Pdots without folate functionalization. The scale bars are 30 μm. Reproduced with permission [44]. Copyright: 2015, American Chemical Society.

Liu et al. [44] employed the same technique for streptavidin immobilization on CPNPs of the similar quinoxaline based polymer **36**. These CPNPs showed the same selectivity for the imaging of microtubules in HeLa cells and the imaging of MCF-7 cell membranes. In addition, they utilized receptor mediated endocytosis to label ovarian cancer cells with overexpressed folate receptors (SKOV-3 cells). This was possible after surface functionalization of the Pdots with folic acid. Strong red fluorescence was observed for cells stained with folate functionalized Pdots, while a significantly lower intensity red fluorescence was observed for cells treated with the bare, non-functionalized Pdots (Figure 7).

The introduction of carboxylic acid groups can also be achieved through hydrolysis of ester groups present on the polymer side chains. This technique was utilized by Peters et al. [41], who prepared PPV based (**4**, Figure 4) CPNPs with ester functionalities. The ester groups were hydrolyzed during the washing steps following particle formation. As a proof-of-concept, they immobilized a gold-labeled antibody to the NP surface by means of EDC coupling. The success of the reaction was shown by TEM and was confirmed by energy-dispersive X-ray (EDX) spectroscopy.

Functional groups that directly link with receptors on the cells of interest can also be introduced onto the CP during polymer synthesis. Liu et al. [54] introduced phenylboronic acid (PBA) groups on a poly(fluorene-*alt*-benzothiadiazole) copolymer (**37**). PBA is known to undergo a pH driven reversible esterification reaction with *cis*-diol compounds to form cyclic boronates. This reaction can be of interest for the selective targeting of sialic acid (SA, a 9-C monosaccharide) overexpressed cancer cells like DU-145 (prostate cancer cell line). Unfortunately, PBA has no preference for SA over other monosaccharides. This problem was solved by SA-template imprinting in the CPNPs during co-precipitation. SA was subsequently removed by adjusting the pH followed by dialysis. The cavities formed on the surface of the CPNPs perfectly fit SA, leading to a selective targeting of cancer cells with SA overexpression.

**Figure 8:**
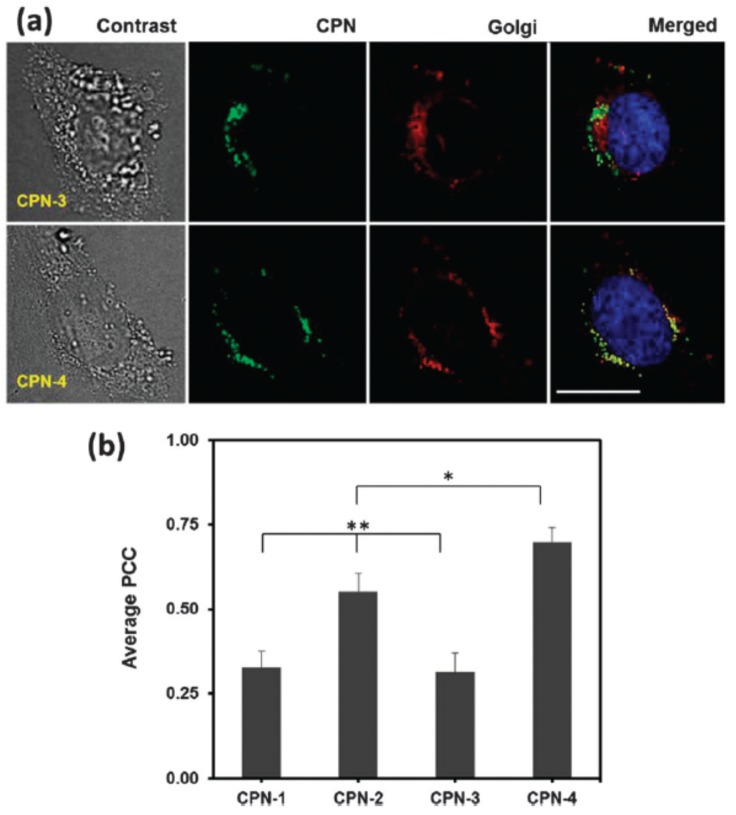
(**a**) Microscopic images of HeLa cells incubated with CPNP-3 and CPNP-4, followed by Golgi (red) and nucleus (blue) staining. The scale bar is 20 µm. CPNP-4 exhibits a higher overlap with Golgi than CPNP-3; (**b**) quantitative analysis of co-localization using the PCC (Pearson’s correlation coefficient) algorithm. Co-localization with Golgi is dependent on the side chain and backbone structures. The error bar represents ±standard deviation (*n* = 3). * <0.05 when CPNP-4 compared with CPN-2. ** <0.0005 when CPNP-1 and CPNP-3 are compared with CPNP-2 and CPNP-4 (*n* = 3). Reproduced with permission [55]. Copyright: 2013, Royal Society of Chemistry.

Another strategy was employed by Mendez et al. [55], who obtained subcellular localization of PPE-based CPNPs (**38**–**41**, denoted as CPNP-1 to CPNP-4, respectively) driven by the type of functional groups on the CP side chains. The presence of primary amine groups (CPNP-2) and a higher flexibility of the polymer backbone (CPNP-4) increased Golgi localization, whereas the presence of short ethylene glycol side chains (CPNP-1) and tertiary amine groups (CPNP-3) decreased Golgi localization (Figure 8). Moreover, the cell cytotoxicity for CPNP-1 and CPNP-3 was higher.

**Figure 9:**
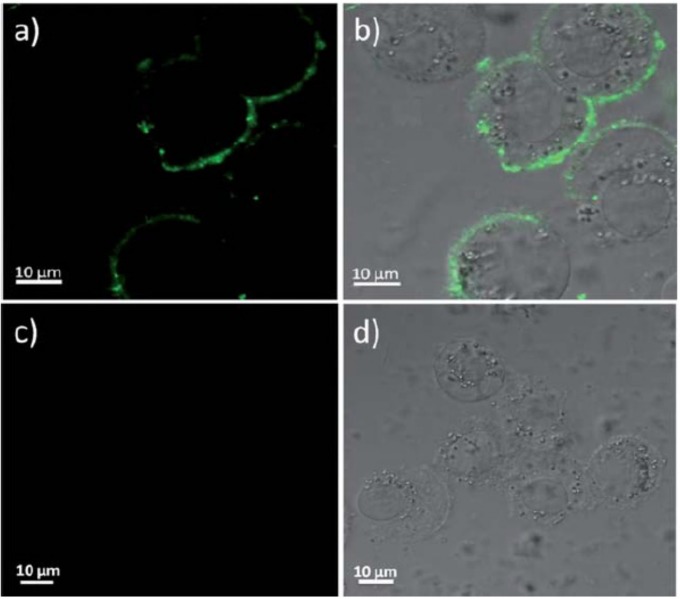
Confocal fluorescence images of HT-29 cells labeled with cyclic RGDfK tagged PEG-PFBD **42** dots after 15 min incubation at room temperature (top row) and non-functionalized PEG-PFBD dots (bottom row). Scale bar: 10 μm. Reproduced with permission [56]. Copyright: 2012, Royal Society of Chemistry.

Sometimes, functional groups are not directly bound to the CP, but to surfactant or matrix molecules. Li et al. [56] prepared CPNPs from poly(fluorene-*co*-benzoxadiazole) **42** via the mini-emulsion technique, using PEG-COOH as the surfactant. Remarkably, small CPNPs were formed, with a hydrodynamic diameter of only 20 nm. No broadening of the absorption spectra of the CPNPs of **42** in water compared to the molecularly dissolved polymer (in dichloromethane) was observed. This indicates a low amount of inter-chain aggregates in the particles, which can explain the exceptionally high PLQY of 46%. Bioconjugation with a cyclic amine labeled RGDfK peptide was achieved in the presence of EDC and *N*-hydroxysulfosuccinimide (sulfo-NHS). After incubation of HT-29 human colon cancer cells, the particles were found to clearly bind to the cells, whereas, for the non-labeled NPs, no fluorescence was observed (Figure 9).

Feng et al. [57] developed four different CPNPs with four different colors. To achieve this, they co-precipitated fluorene based alternating copolymers **43**–**46** with poly(styrene-*co*-maleic anhydride) (PSMA). After particle formation, modification of the surface with carboxyl groups was conducted. Different antibodies were then successfully immobilized onto the particle surfaces via amide coupling. This variety in functionalized CPNPs enabled a double-antibody recognition mode for the specific detection of cancer cells. In this work, differentiation between SK-BR3, MCF-7 and HeLa cells was demonstrated. One batch of CPNP45 was functionalized with anti-EpCAM and another with anti-ErbB2. Treatment of SK-BR3, MCF-7 and HeLa cells with the particles was performed and only SK-BR3 and MCF-7 cells were stained with the anti-EpCAM particles, while the anti-ErbB2 particles only stained the HeLa cells. All three cell types could be distinguished, even though the SK-BR3 and MCF-7 belong to the same breast cancer cell lines.

The introduction of β-cyclodextrin (CD) units on the outer ends of polyfluorene (**47**) chains was investigated by Sun et al. [58]. They modified the CPNPs via the typical host–guest interaction between CD and adamantine (ADA), which was introduced on the chain ends of four different glycopolymers. The glycopolymer functionalized NPs (Lac-NP) showed an excellent binding to lectines like galectin-3 (GAL), which has been shown to be crucial in cell–cell interactions related to many diseases like cancer. Gal-NP specifically entered Hep-G2 cells (liver cancer cells) that express the Gal-specific ASGP receptor on their surface, which enables specific targeting of turmeric tissue.

Copper catalyzed azide-alkyne click (CuAAC) functionalities are bio-orthogonal, indicating that no reactions occur between those functional moieties and bio-available functional groups. This is a remarkable asset compared to regularly employed functional groups such as carboxylic acids or amines that are reactive in living cells. The inert nature of those bio-orthogonal groups generally makes them less cytotoxic than for example NHS-coupled counterparts and non-specific adsorption is often reduced as well. Li et al. [59] developed hybrid CPNPs consisting of poly(fluorene-*co*-phenylene) **48** (PFP) combined with an azide-functionalized PEG-chain (PLGA-PEG-N_3_). Because of the hydrophobic nature of the PFP and the hydrophilicity of PLGA-PEG-N_3_, a fluorescent PFP core is formed and the azide functionalities are pointing outwards due to the hydrophilic nature of the PEG. Plerixafor (PLE) is an FDA (U.S. Food and Drug Administration) approved drug, known to inhibit endocytosis of CXCR4 transmembrane proteins, which makes it the ideal ligand for cell membrane labelling. Alkyne functionalities were introduced onto PLE and a CuAAC reaction was performed between the CPNPs and PLE. The CPNPs were found to effectively locate on the cell membrane.

CPNPs embedded in a covalently bound hydroxyl-containing matrix material were developed by Zhou et al. [60] through the introduction of click functionalities. By copper-free thermally initiated click chemistry, they were able to form NPs from azide functionalized fluorene-*alt*-benzothiadiazole copolymer **49** and an alkyne-functionalized hyperbranched polyglycerol (HPG). The size of the particles could be adjusted from 40 to 210 nm and PLQYs up to 23% were obtained. MCF-7 breast cancer cells were treated with the particles and efficient internalization was observed. Unfortunately, no bioconjugation of the CPNPs was shown.

**Figure 10:**
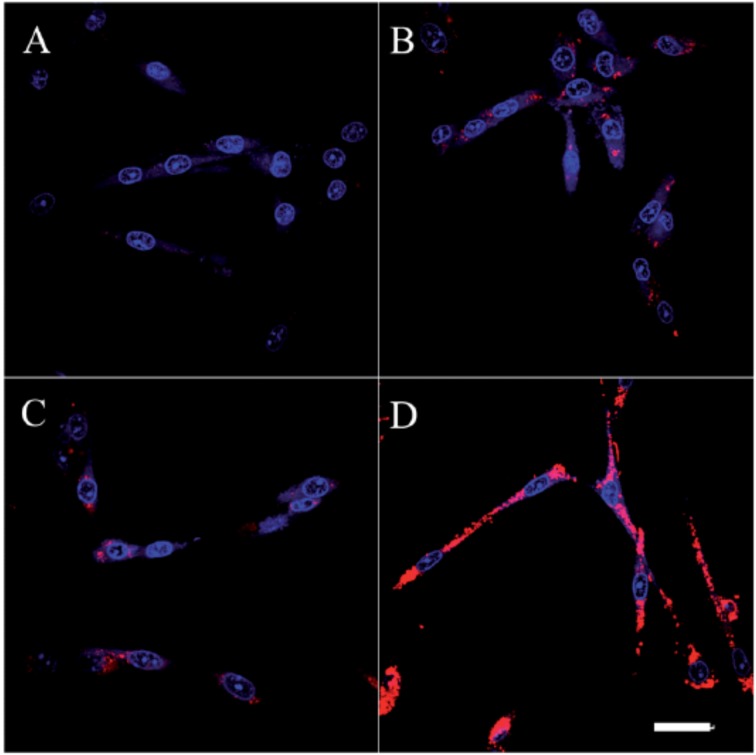
Confocal laser scanning microscopy (CLSM) images of fixed SKBR-3 breast cancer cells incubated with CPNP**14**-Mal (**A**); CPNP**15**-Mal (**B**); CPNP**14**-Affibody (**C**) and CPNP**15**-Affibody (**D**) at 37 °C overnight. All images share the same scale bar of 30 µm. Reproduced with permission [45]. Copyright: 2013, Royal Society of Chemistry.

Liu et al. [45,46] designed CPNPs synthesized from polymers **14**, **15** and **16** (Figure 4) in a matrix of 1,2-distearoyl-*sn*-glycero-3-phosphoethanolamine-*N*-[methoxy(polyethyleneglycol)-2000] (DSPE-PEG_2000_) and its maleimide modified derivative DSPE-PEG_2000_-Mal. A hydrophobic core consisting of the conjugated polymer (**14**, **15** or **16**) was formed, while the hydrophilic PEG and PEG-Mal point outwards. Click functionalization of the maleimide groups with anti-HER2 affibody (for **14** and **15** based CPNPs) or human immunodeficiency virus type 1 (HIV-1) trans-activating transcriptional activator (Tat, for **16** based CPNPs) was performed to increase the HER2-overexpressed SKBR-3 breast cancer cell (CPNP**14** and CPNP**15**) or HepG2 liver cancer cell (for CPNP**16**) internalization efficiency compared to the non-functionalized CPNPs. The fluorescence intensity of SKBR-3 cells incubated with affibody functionalized and non-functionalized CPNP**14** and CPNP**15** was compared (Figure 10). Few non-bioconjugated particles are able to enter the cancer cells due to their PEG shell, which inhibits nonspecific cellular internalization (Figure 10A,B), while, for CPNP**14**-Mal, no fluorescence could be observed, and some weak fluorescent dots did appear for CPNP**15**-Mal. A strong increase in fluorescence brightness was observed for CPNP15-Affibody (Figure 10D). For both, the increase in emission can be ascribed to the remarkably higher PLQY of the CPNPs formed by the POSS-functionalized polymer **15**. The introduction of affibody on the CPNPs surface induces specific cell uptake of the particles (compare Figure 10C and Figure 10A,B,D).

**Figure 11:**
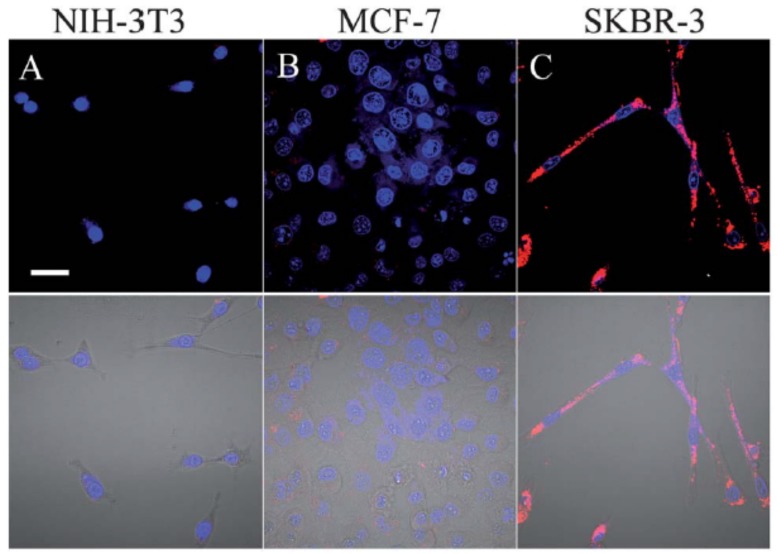
CLSM fluorescence (top) and fluorescence/transmission overlay (bottom) images of fixed NIH-3T3 (**A**); MCF-7 (**B**) and SKBR-3 (**C**) cells incubated overnight with 5 nM CPNP**15** at 37 °C. All images share the same scale bar of 30 µm. Reproduced with permission [45]. Copyright: 2013, Royal Society of Chemistry.

**Figure 12:**
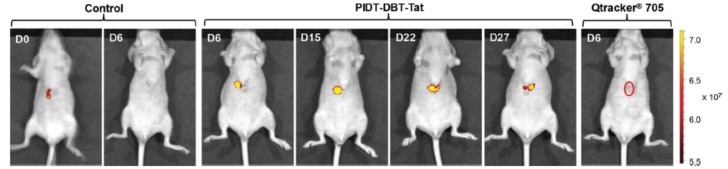
Representative in vivo fluorescence images of a mouse transplanted with 4 × 10^6^ of HepG2 cells labeled by CPNP**16** and Qtracker 705. Control images were obtained from a nude mouse that underwent the same surgical operation without injection of labeled HepG2 cells. The images were taken on designated days post cell injection (λex = 640 nm, 720/20 nm filter). Reproduced with permission [46]. Copyright: 2015, Wiley.

The same research group also showed that the affibody-bioconjugated CPNP**15**s were able to specifically enter HER2-overexpressed breast cancer cells (SKBR-3), while no uptake in non-HER2-overexpressed breast cancer cells (MCF-7) and normal cells (MIH-3T3 fibroblasts) could be observed (Figure 11) [45]. With CPNP**16**, in vivo monitoring of liver tumor growth was possible up to 27 days because of the long residence time of the particles in the body (Figure 12) [46].

Koner et al. [61] co-precipitated commercially available polymers **50** and **51** with polystyrene (PS) decorated with either hydroxyl or carboxylic acid terminated PEG side chains. The hydrophobic PS backbone entangles in the hydrophobic CP core of the particles, while the functionalized PEG chains point outwards. The particles exhibited an excellent PLQY of 57% and high stability. After particle formation, the hydroxyl groups were activated with 1,1′-carbonyldiimidazole (CDI), which allows further reactions with amines to form carbamates. Particle functionalization with streptavidin was performed and HeLa cervical cancer cells were incubated with biotin and the carboxylic acid or hydroxyl functionalized CPNPs. Biotinylated cells were clearly marked by the fluorescent Pdots. Non-biotinylated cells were non-specifically bound by the carboxylic acid functionalized particles, whereas no binding was observed for the hydroxyl terminated particles. The introduction of hydroxyl groups on the CPNP surface thus reduces non-specific cell binding.

Folic acid receptors are over-expressed on MCF-7 breast cancer cells. However, the introduction of folic acid directly onto the polymer side chains increases the synthetic complexity of CPNPs and prevents post-NP-formation functionalization reactions. Ding et al. [48] prepared functionalized CPNPs via co-precipitation of polymer **20** (Figure 4) with DSPE-PEG_2000_ and FA-functionalized PEG (DPSE-PEG_5000_-FA). MCF-7 breast cancer cells with overexpressed FR and NIH/3T3 fibroblast normal cells with low FR expression were incubated with the FA-functionalized and non-functionalized Pdots. A remarkably more intense fluorescence (×1.8) could be observed for the MCF-7 cells treated with the FA-functionalized particles, showing that active transport of the NPs via FR is occurring. Furthermore, when free FA is available in the medium, less NP internalization in the MCF-7 cells was observed, indicating that the FA groups on the particle surface mediate cell entrance. The functionalized and non-functionalized NPs exhibited an equal fluorescence intensity after NIH/3T3 staining due to the low FR expression in those cells.

The introduction of amphiphilic peptides as capping ligands was demonstrated by Almeida et al. [62]. Three different peptide sequences were conjugated to a branched aliphatic chain by their N-terminus, enabling embedding of the aliphatic tail in the CP core (**50**), while the peptide sequences are exposed to the medium. The first sequence was the positively charged cell penetrating TAT sequence (2-hexyldecane-GRKKRRQRRRPQ-amide), the second one the negatively charged anti-TAT sequence (2-hexyldecane-GDEEDDQDDDPQ-amide, designed to mimic the TAT sequence) and the third a zwitterionic PEK peptide (2-hexyldecane-PPPPEKEKEKEK-amide), which is known to inhibit cellular uptake. HeLa cells were treated with the peptide-functionalized particles (TAT/NP, anti-TAT/NP and PEK/NP) and after 30 min, internalization of TAT/NP into the perimembraneous region was observed. After 2 h of incubation, the particles migrated to the cytoplasmic region, whereas, after 24 h, accumulation in the perinuclear region was noticed. The anti-TAT/NP and PEK/NP showed minimal cell internalization due to the negative charges on the peptide chains. Furthermore, they showed altered emission colors through the use of other CPs, although the cellular uptake of the particles remained the same. Via this multi-color imaging, Almeida and coworkers were able to point out that cell internalization of negatively charged particles was facilitated by TAT/NPs when cells were treated with both particles at the same time.

Non-polymeric materials used as encapsulators can often be functionalized in a more straightforward manner. Joshi et al. [63] encapsulated PPV-based (**52**) CPNPs with a porous silica matrix to increase the stability of the particles. Silica is a well-known carrier that is biocompatible, non-interfering, transparent to visible light and easy to functionalize. Modification of the silica coated PPV-CPNPs was achieved by coating the particles with (3-aminopropyl)triethoxysilane (APTES), an amine modified silane layer. After modification, the zeta-potential of the particles rose from −41 to 18 mV, indicating that the surface hydroxyl groups are exchanged by amine groups pointing outwards. Covalent linking of ligands onto the particles was not shown in this work.

### 2.5. Theranostic CPNPs

**Figure 13:**
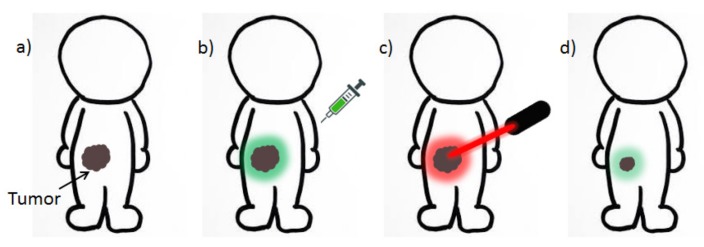
Cartoon-like visualization of the basic concepts of theranostics: (**a**) a patient with cancer is administered a theranostic agent, which is used to visualize the tumor (**b**) and, at the same time, can be activated by a laser to produce reactive oxygen species or heat to damage the cancer cells (**c**). The theranostic NPs can also be loaded with a drug, which is released at the tumor site. Tumor reduction can be observed in situ because of the fluorescent nature of the agent (**d**).

**Figure 14:**
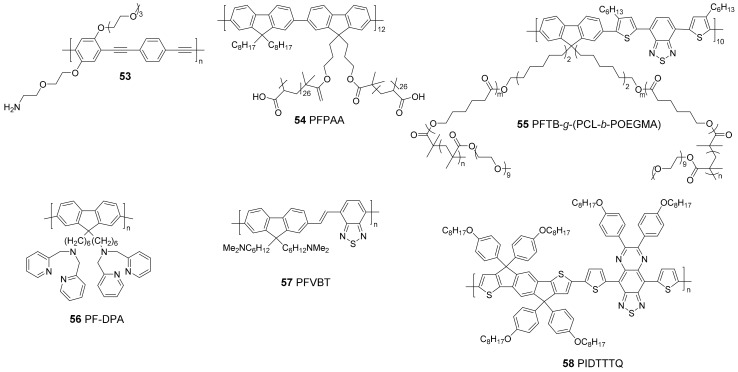
CPs used to form theranostic CPNPs.

Theranostic agents combine diagnosis and therapy (Figure 13). The theranostic properties of CPNPs have been investigated in multiple cases, wherein the fluorescence brightness of the particles leads to diagnosis (e.g., cancer) due to specific binding of the CPNPs to specific receptors (e.g., FR). When the particles are loaded with a drug, it can immediately be delivered and released at the site of interest. On the other hand, controlled and located release of the pharmaceuticals can also decrease the toxicity to healthy cells. The CP structures discussed in this section are gathered in Figure 14.

Chen et al. [64] developed self-assembled NPs from the amphiphilic PPE copolymer **53**. The hydrophobic CP backbone constitutes the core of the particles, while the hydrophilic triethylene glycol monomethyl ether side chains and the amine groups will point outwards. Internalization of the CPNPs in human prostate cancer cells (PC3) was illustrated and the cell cytotoxicity was found to be low. Because of the rigid and hydrophobic nature of the polymer backbone, it was possible to load the particles with the anti-cancer drug doxorubicin (DOX), which is internalized via strong intermolecular π–π stacking interactions. A loading capacity of 5.1% was achieved. In vitro drug release studies were performed by staining of the PC3 cells with the loaded CPNPs. After particle internalization, slow DOX release was observed. Furthermore, cell viabilities decreased with increasing DOX loading. It should be noted that the loaded CPNPs had a lower cytotoxicity to the PC3 cells than free DOX at the same dose due to the prolonged release of DOX from the PPE-NPs. This is also reflected in the IC_50_ (half maximal inhibitory concentration) value for the PPE-NPs (4.23 µg/mL), which is higher than the value for free DOX (1.71 µg/mL). Overall, the growth of cancer cells could be inhibited by the use of the loaded CPNPs, while the toxicity of the drug could be reduced.

Lu et al. [65] synthesized fluorene copolymer **54**, a grafted CP with multiple carboxylate groups on the brush-like side chains. These carboxylic acid groups served as a polydentate ligand to bind and thus stabilize magnetic Fe_3_O_4_ nanoparticles (MNPs). The CP is highly fluorescent, leading to MNPs with a PLQY of 21%, which is boosted by the brush-like polymer structure, preventing inter-chain aggregation and thus fluorescence quenching. The superparamagnetic properties of the particles enable imaging via MRI and the delivery of the particles to target sites via an external applied magnetic field can be performed while monitoring the process with a fluorescent microscope. Low cytotoxicity of the MNPs was observed after treatment of NIH-3T3 fibroblasts. Due to the brush-like structure of **54**, more DOX molecules can be accommodated, leading to high drug loadings of 10 wt %. BGC-823 human gastric cancer cells were incubated with the DOX-loaded MNPs and a good therapeutic efficiency was observed. After 10 h, only 30% of the cancer cells survived, while a 90% cell viability of the cells treated with DOX-free particles was noted. Furthermore, drug release was shown to be pH dependent, with better results in a more acidic environment. This is beneficial for therapeutic use in cancer cells because their cytoplasm is slightly acidic.

The pH dependent DOX release was also observed by Yang et al. [66] They synthesized a poly(fluorene-*alt*-benzothiadiazole) copolymer (**55**) grafted with PCL and poly[oligo(ethylene glycol) methyl ether methacrylate] (POEGMA) block copolymers. Those bottlebrush-like polymers form highly fluorescent unimolecular micelles, with a PLQY up to 25% through prevention of intermolecular aggregation. The influence of the presence of the PCL block on the PLQY was investigated and an increase of 5% was observed, from 17 to 22%. Furthermore, the PCL block serves as a reservoir for DOX loading. The longer the PCL block, the more DOX could be loaded. Amounts up to 10 wt % could be achieved. On the other hand, DOX release was dependent on the POEGMA chain length, with an optimal degree of polymerization of 29. Longer chains retarded the DOX release due to their bulkiness. The DOX-loaded unimolecular micelles showed a low cytotoxicity for normal cells (L929), whereas the toxicity for cervical cancer cells (HeLa cells) was remarkably higher. This effect could be attributed to the pH dependent DOX release.

Muthuraj and co-workers developed polyfluorene based (**56**) CPNPs with dual state emission, but rather low PLQY (3%) [67]. These particles have the unique property of being toxic to melanoma (B16F10) and ovarian cancer (SKOV-3) cells, even without drug loading. On the other hand, the cytotoxicity to normal cells (NIH-3T3 and CHO cells) is minimal. These results were reflected in the IC_50_ values obtained for NIH-3T3 (>2000 µg/mL) and CHO cells (1851 µg/mL), which were higher than those of B16F10 (411 µg/mL) and SKOV-3 cells (766 µg/mL). A clear inhibition of cancer cell proliferation was observed, even for low concentrations (<200 µg/mL). This observation can be ascribed to a larger particle uptake in cancer cells compared to normal cells under the same treatment concentrations. The di(picolyl)amine (DPA) functionalities on the polymer side chains have been shown to trigger the formation of reactive oxygen species (ROS), which play a crucial role in cancer cell death. More ROS were produced in B16F10 cancer cells treated with the CPNPs compared to non-treated cells, leading to more cell death. Due to the multifunctional nature of the particles, no leaking of the highly toxic anti-cancer drug out of the particles was observed, preventing severe side effects for the patients to be treated.

**Figure 15:**
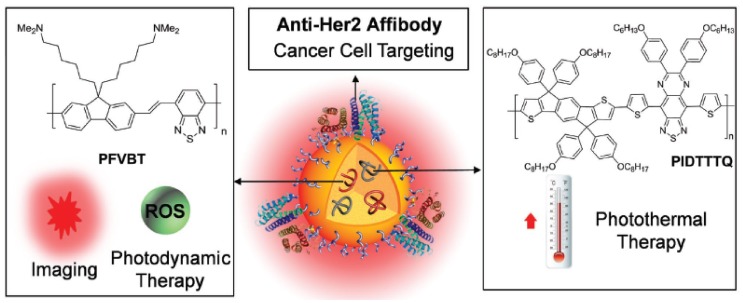
Schematic illustration of the components and functions of anti-HER2-CPNPs consisting of **57**, **58** and DSPE-PEG_2000_-maleimide. Reproduced with permission [68]. Copyright: 2017, Wiley.

**Figure 16:**
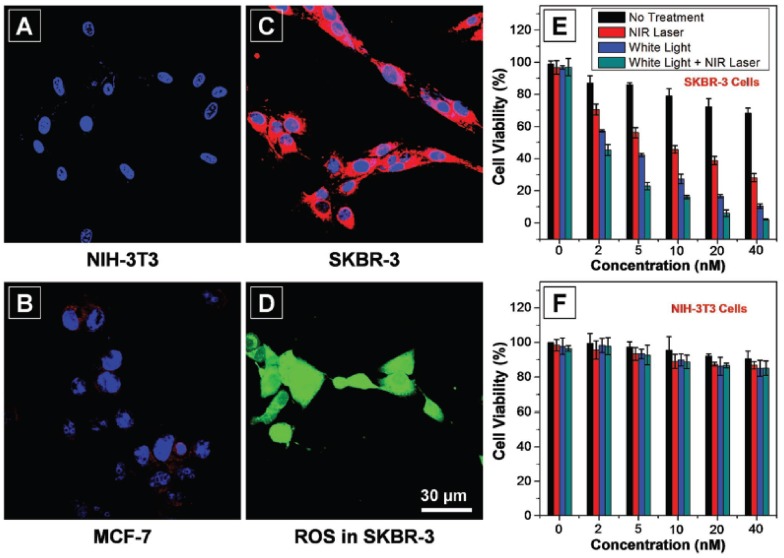
Confocal images of (**A**) NIH-3T3; (**B**) MCF-7; and (**C**) SKBR-3 cells after 4 h incubation with anti-HER2-CPNPs (2 × 10^−9^ M). The red fluorescence of anti-HER2-CPNPs is collected above 505 nm upon excitation at 488 nm. The blue fluorescence of Hoechst from the nucleus is collected from 430 to 470 nm upon excitation at 405 nm; (**D**) detection of intracellular ROS generation by DCF-FA in SKBR-3 cells after incubation with anti-HER2-CPNPs (2 × 10^−9^ M, 4 h) followed by light irradiation (30 s). Images (**A**–**D**) share the same scale bar of 30 μm. Cell viabilities of (**E**) SKBR-3 and (**F**) NIH-3T3 cells after incubation with anti-HER2-CPNPs (2 × 10^−9^ M, 4 h) followed by photodynamic and photothermal treatment. Reproduced with permission [68]. Copyright: 2017, Wiley.

Multifunctional CPNPs consisting of two different CPs (**57** and **58**) in a DSPE-PEG_2000_-maleimide matrix were very recently developed by Feng et al. (Figure 15) [68]. PFVBT **57** is highly fluorescent, leading to a PLQY of 23% for the formed CPNPs. Moreover, it can also efficiently transform light into ROS. On the other hand, PIDTTTQ **58** is a non-fluorescent material that can convert light into thermal heat, which makes the particles suitable for photothermal therapy. Since the CPNPs are toxic to biological tissue under irradiation, specific particle localization is of utmost importance. This was achieved by click immobilization of anti-HER2 affibody onto the particle surface. CPNP internalization was only observed for HER2 overexpressed SKBR-3 breast cancer cells, while no uptake was noticed for MCF-7 breast cancer cells and NIH-3T3 normal fibroblast cells lacking HER2 expression (Figure 16). Successful tumor cell (SKBR-3) death was observed after incubation with the CPNPs and irradiation with NIR laser light and/or white light (Figure 16E), which can partly be ascribed to ROS formation. This was demonstrated by the cell permeable fluorescent dye dichlorofluorescein diacetate (DCF-DA), an ROS indicator that is rapidly oxidized to dichlorofluorescein (DCF), affording a bright green fluorescence (Figure 16D).

Finally, Gesquiere and coworkers used MEH-PPV (see Figure 6) as a photosensitizer for photodynamic therapy [69,70]. Due to its high extinction coefficient and easy intersystem crossing to the triplet state, ROS are readily generated. For specific targeting of cancer cells with overexpressed folate receptors (OVCAR3), blended MEH-PPV NPs with amphiphilic PS-PEG-COOH were formulated. The NPs could be functionalized with folic acid through the carboxylic acid groups. The functionalized particles were found to exhibit no dark cytotoxicity and appeared selective for the OVCAR3 cell line, resulting in near complete cell death.

## 3. Conclusions

Conjugated polymer nanoparticles are attractive fluorescent bioimaging probes due to their excellent optical properties and low cytotoxicity. Up until now, a whole range of conjugated polymer particles has been synthesized, with particle sizes ranging from the nano- to the micrometer scale and with colors covering the entire visible range. Most conjugated polymers used for imaging have a rather simple backbone structure, often based on fluorine [40,42,43,44,45,48,57,60,61]. On the other hand, a huge variety of push–pull type semiconducting polymers have recently been studied for optoelectronic applications, notably organic photovoltaics [71]. We believe that many of these materials could be useful for imaging too. The combination of strong donor and acceptor monomers leads to low bandgap materials of which the absorption and emission maxima can be pushed toward the NIR region. This wavelength range is of particular interest for bioimaging because of the low autofluorescence and deep tissue penetration of NIR light. Unfortunately, the photoluminescence quantum yield generally drops when the NIR regime is approached. Different techniques have been employed to boost the fluorescence intensity. Most of these techniques rely on the prevention or limitation of π–π stacking in the particles. Nevertheless, quantum efficiencies hardly reach values over 50%. The introduction of specific moieties known to exhibit aggregation induced emission seems to be an attractive alternative solution, since those materials exhibit higher emission efficiencies when tight packing can be achieved [40,43,44,45,46,47,48,49,50,51].

Surface functionalization of the conjugated polymer nanoparticles is needed to target specific cells. Functional groups allowing bioconjugation have been introduced on the conjugated polymer backbone itself or on non-conjugated (matrix) materials such as PEG chains or silica. The most frequently introduced functional groups are carboxylic acids and amines. However, due to their pH dependent charge, functionalized particles experience difficulties to enter cells under certain conditions. Click functionalities like maleimides, alkynes and azides have been explored as alternatives. A vast range of immobilization methods have been studied so far, allowing the immobilization of nearly any ligand to the particle surface [41,43,44,45,46,48,52,53,54,55,56,57,58,59,60,61,62,63].

Since specific cell targeting can be achieved, conjugated polymer nanoparticles are also of interest for therapeutic applications. The advantage of delivering cell-attacking drugs to the region of interest minimizes healthy cell destruction. Several conjugated polymer nanoparticles were loaded with doxorubicin, a well-known anti-tumor drug, via non-covalent interactions. The side chains of the conjugated polymers in these cases typically act as a reservoir for the drug molecules. Other examples use conjugated polymers known to generate reactive oxygen species, which are toxic for tumor cells [64,65,66,67,68].

On the basis of the steady improvements on the brightness, stability, cell viability, specificity and theranostic nature of many conjugated polymer nanoparticles reported in literature, a ‘bright’ future lies ahead for conjugated polymer nanoparticle bioimaging.

## Figures and Tables

**Table 1:** Overview of conjugated polymer nanoparticles used in bioimaging.

Polymer	NP/Hybrid	Hybrid Material	Preparation Method	Particle Size (nm)	PLQY (%)	Other Applications	Ref.
**30**, **31**	NP	/	Solvent exchange	4–264	14–26	/	[52]
**7**–**9**, **32**	NP	/	Solvent exchange	23	1–36	/	[43]
**38**–**41**	NP	/	Solvent exchange	58–87	/	/	[55]
**47**	NP	/	Solvent exchange	42–57	/	/	[58]
**10**–**12**, **36**	NP	/	Solvent exchange	22	47	/	[44]
**5**, **6**	NP	/	Solvent exchange	16	13, 19	/	[42]
**20**	Hybrid NP	PEG	Solvent exchange	80	27	/	[48]
**52**	Hybrid NP	Silica	Solvent exchange	5–50	1.5	/	[63]
**23**–**26**	NP	/	Solvent exchange	50–100	76	/	[50]
**16**	Hybrid NP	PEG	Solvent exchange	56	/	/	[46]
**2**	Hybrid NP	PEG and dye	Solvent exchange	45	45	/	[40]
**21**, **22**	Hybrid NP	PCL-*b*-POEGMA	Solvent exchange	50–500	26	/	[49]
**14**, **15**	Hybrid NP	PEG	Solvent exchange	28	14	/	[45]
**50**, **51**	Hybrid NP	PS-PEG	Solvent exchange	13	57	/	[61]
**43**–**46**	Hybrid NP	PSMA	Solvent exchange	30	3–78	/	[57]
**57**, **58**	Hybrid NP	DSPE-PEG	Solvent exchange	30	23	Theranostic	[68]
**37**	NP	/	Solvent exchange	30	14	/	[54]
**30**–**32**	NP	/	Solvent exchange	16–21	17–30	/	[52]
**27**–**29**	NP	/	Emulsion polymerization	25–73	56	/	[51]
**53**	NP	/	Self-assembly	117	/	Theranostic	[64]
**56**	NP	/	Self-assembly	24	3	Theranostic	[67]
**1**	NP	/	Mini-emulsion	2.9	1.7	/	[36]
**3**, **4**	NP	/	Mini-emulsion	116, 117	3	/	[41]
**17**–**19**	NP	/	Mini-emulsion	78–188	8–13	/	[47]
**42**	Hybrid NP	PEG	Mini-emulsion	20	46	/	[56]
**48**	Hybrid NP	Azide-funct. PEG	Mini-emulsion	130	4	/	[59]
**49**	Hybrid NP	HPG	Mini-emulsion	40–210	23	/	[60]
**50**	Hybrid NP	Peptide	Mini-emulsion	40	37–42	/	[61]
**54**	Hybrid NP	Fe_3_O_4_	Ligand exchange on Fe_3_O_4_	26	21.5	Theranostic	[65]
**55**	NP	/	1 polymer brush/NP	20–54	20–30	Theranostic	[66]

NP = nanoparticle; PLQY = photoluminescence quantum yield; PEG = polyethylene glycol.
